# Assessment of Metabolic Interaction between Repaglinide and Quercetin via Mixed Inhibition in the Liver: In Vitro and In Vivo

**DOI:** 10.3390/pharmaceutics13060782

**Published:** 2021-05-23

**Authors:** Ji-Min Kim, Seong-Wook Seo, Dong-Gyun Han, Hwayoung Yun, In-Soo Yoon

**Affiliations:** Department of Manufacturing Pharmacy, College of Pharmacy, Pusan National University, Busan 46241, Korea; jiminkim@pusan.ac.kr (J.-M.K.); sswook@pusan.ac.kr (S.-W.S.); hann9607@pusan.ac.kr (D.-G.H.)

**Keywords:** drug-phytochemical interaction, hepatic metabolism, mixed inhibition, quercetin, repaglinide

## Abstract

Repaglinide (RPG), a rapid-acting meglitinide analog, is an oral hypoglycemic agent for patients with type 2 diabetes mellitus. Quercetin (QCT) is a well-known antioxidant and antidiabetic flavonoid that has been used as an important ingredient in many functional foods and complementary medicines. This study aimed to comprehensively investigate the effects of QCT on the metabolism of RPG and its underlying mechanisms. The mean (range) IC_50_ of QCT on the microsomal metabolism of RPG was estimated to be 16.7 (13.0–18.6) μM in the rat liver microsome (RLM) and 3.0 (1.53–5.44) μM in the human liver microsome (HLM). The type of inhibition exhibited by QCT on RPG metabolism was determined to be a mixed inhibition with a K_i_ of 72.0 μM in RLM and 24.2 μM in HLM as obtained through relevant graphical and enzyme inhibition model-based analyses. Furthermore, the area under the plasma concentration versus time curve (AUC) and peak plasma concentration (C_max_) of RPG administered intravenously and orally in rats were significantly increased by 1.83- and 1.88-fold, respectively, after concurrent administration with QCT. As the protein binding and blood distribution of RPG were observed to be unaltered by QCT, it is plausible that the hepatic first-pass and systemic metabolism of RPG could have been inhibited by QCT, resulting in the increased systemic exposure (AUC and C_max_) of RPG. These results suggest that there is a possibility that clinically significant pharmacokinetic interactions between QCT and RPG could occur, depending on the extent and duration of QCT intake from foods and dietary supplements.

## 1. Introduction

**Figure 1:**
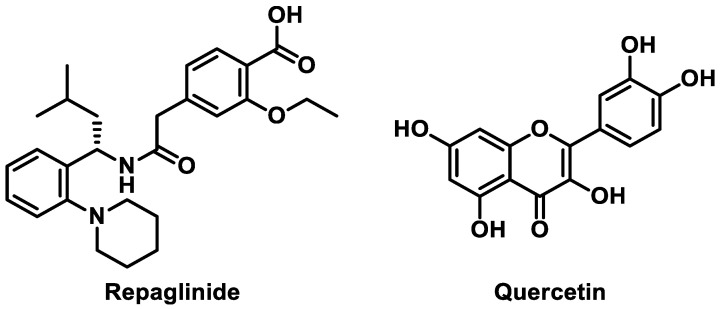
Chemical structures of repaglinide and quercetin.

Repaglinide (RPG; Figure 1), a rapid-acting meglitinide analogue, is an oral hypoglycemic agent for patients with type 2 diabetes mellitus [1]. It lowers postprandial blood glucose levels by promoting insulin secretion from pancreatic β-cells [2]. RPG reduces the risk of hypoglycemia by stimulating insulin secretion only when blood glucose levels are higher than normal, whereas sulfonylureas induce insulin secretion even at low blood glucose levels [3,4]. Additionally, treatment with RPG has been shown to improve oxidative stress indices in type 2 diabetic patients, potentially reducing the risk of diabetes-associated vascular disease [5]. The oral absorption of RPG is rapid and complete but its bioavailability is low because of considerable first-pass metabolism [6]. RPG is primarily eliminated via cytochrome P450 (CYP)-mediated oxidative metabolism in the liver; in particular, both CYP2C8 and CYP3A4 are the principal CYP isoforms responsible for the biotransformation of RPG [7,8].

Over the past decades, bioactive flavonoids from various medicinal herbs and dietary supplements have gained increasing interest because of their important roles in complementary and alternative medicines [9,10]. A previous literature review of 18 studies over 9 countries indicated that the prevalence of complementary and alternative medicine use among people with diabetes ranges from 17% to 72.8% [11]. Quercetin (QCT; Figure 1), one of the most extensively explored flavonoids, is commonly found in many fruits, vegetables, and grains [12,13]. QCT is a widely recognized nutraceutical commercially available in capsule and tablet forms, consumed at a daily dose of 1 g or more [14]. It is a potent antioxidant and anti-inflammatory phytochemical that exerts a wide range of protective and therapeutic activities against arthritis, cancer, cardiovascular disease, diabetes, neurodegenerative disease, and obesity [15]. In particular, the mechanisms of the antidiabetic action of QCT include the inhibition of intestinal glucose absorption, stimulation of insulin secretion, and enhancement of peripheral glucose utilization, which contribute to improving whole-body glucose homeostasis [16]. Thus, there is a possibility that QCT can be used as a complementary medicine concurrently with RPG for the prevention and treatment of type 2 diabetes.

QCT is eliminated mainly through phase II metabolism, such as glucuronidation, sulfation, and methylation in the liver [17]. QCT has been used as a selective CYP2C8 inhibitor in CYP phenotyping studies [18,19,20]; however, some reports have shown the inhibitory effects of QCT on other CYP isozymes [21,22]. In particular, a previous study revealed that QCT profoundly inhibited the activity of several CYPs including CYP2C8 and CYP3A4 [23], while another more recent study reported that QCT significantly inhibited CYP3A4 activity with a K_i_ of 15.4 μM [24]. Furthermore, it is important to note that several studies have reported significant drug interactions of RPG with CYP2C8 and CYP3A4 inhibitors. Systemic exposure to orally administered RPG was significantly increased by CYP2C8 inhibitors such as trimethoprim (1.6–2-fold) and clopidogrel (3.1–5.1-fold) [25,26] and by CYP3A4 inhibitors such as telithromycin (1.8-fold) and itraconazole (1.4-fold) [27,28]. Indeed, a previous study reported that QCT (25 μM) inhibited the in vitro metabolism of RPG (0.2 μM) by 58% in human liver microsome (HLM) [8], and another study reported the K_i_ of 0.61 μM for the inhibitory effect of QCT on in vitro metabolism of RPG in HLM [29]. Thus, there is a possibility of in vivo herb–drug interactions between QCT and RPG, but relevant information is currently lacking. This strongly suggests an immediate need for further investigation on this issue to avoid adverse reactions and optimize drug therapy.

Therefore, the present study aimed to comprehensively investigate the effects of QCT on the metabolism and pharmacokinetics of RPG. The inhibitory effect of QCT on the metabolism of RPG and its mechanisms were studied in HLM and rat liver microsomes (RLM). Next, the in vivo pharmacokinetic interactions between QCT and RPG were evaluated in a rat model. The protein binding and blood distribution of RPG were also examined.

## 2. Materials and Methods

### 2.1. Materials

RPG (purity > 98%), QCT (purity ≥ 95%), and ketoconazole (used as an internal standard; purity ≥ 98%) were purchased from Tokyo Chemical Industry Co. (Tokyo, Japan) Ethanol, dimethyl sulfoxide, polyethylene glycol 400 (PEG 400), carboxymethyl cellulose (CMC), potassium phosphate monobasic/dibasic, β-Nicotinamide adenine dinucleotide phosphate (NADPH), and phosphate-buffered saline (PBS) were purchased from Sigma-Aldrich (St. Louis, MO, USA). Pooled male Sprague-Dawley rat plasma and pooled male human plasma were purchased from Innovative Research, Inc. (Novi, MI, USA) Pooled HLM and RLM were purchased from BD-Genetech (Woburn, MA, USA).

### 2.2. Protein Binding and Blood Distribution Studies

The unbound fractions of RPG and QCT in plasma (f_uP_) and hepatic microsomes (f_uMIC_) were measured through an equilibrium dialysis method using a rapid equilibrium dialysis (RED) device (Thermo Fisher Scientific, Inc., Waltham, MA, USA) as described previously [30,31,32,33]. The microsomes and 20-fold diluted plasma were spiked with either RPG alone or in combination with QCT yielding final concentrations of 5 μM for both compounds. A 200 μL spiked samples and 400 μL PBS were placed into the “sample” and “buffer” chambers of the RED device, respectively. After 4-h incubation to equilibrate between the buffer and plasma compartments, the RED plate was sampled from both compartments. The samples were matrix-matched for analysis by addition of either diluted plasma or buffer; (a) blank diluted plasma added to the buffer samples and (b) blank buffer added to the diluted plasma sample, at a ratio of 50:50 *v*/*v*. The unbound fractions in microsomes and diluted plasma were determined by dividing the analyte/IS peak area ratios of the (a) sample by those of the (b) sample. The unbound fraction in diluted plasma (f_u,d_) was converted to the unbound fraction in undiluted plasma (f_u_) using a Kalvass equation as below (DF: dilution factor) [33].

(1) $$f_{u}=\frac{1/\mathrm{DF}}{[(1/f_{u,d})-1]+1/\mathrm{DF}}$$

The blood-to-plasma concentration ratio (R_B_) of RPG was determined as described previously [34]. Briefly, 1 mL of fresh blood was spiked with either RPG alone or in combination with QCT, yielding a final concentration of 5 μM for both compounds, and then incubated at 37 °C for 60 min. A plasma sample was obtained by centrifugation of the blood sample at 2000× *g* for 5 min. The concentrations of RPG in 50 μL of the plasma samples were determined using a validated high-performance liquid chromatography (HPLC) method.

### 2.3. In Vitro Microsomal Metabolism Study

The concentration-dependent disappearance of RPG in the RLM and HLM was evaluated to investigate the kinetics of the hepatic CYP-mediated metabolism of RPG. A microsomal reaction mixture consisting of microsomes (0.3 mg/mL), 1 mM NADPH, 50 mM phosphate buffer, and 1–500 μM RPG in distilled water (DW) was prepared, at a total volume of 200 μL. At 0, 30, and 60 min after starting the metabolic reaction, a 50 μL aliquot of the incubation mixture was sampled and transferred to a clean 1.5-mL microcentrifuge tube containing 150 μL of ice-cold acetonitrile to terminate the metabolic reaction. Following centrifugation at 15,000× *g* for 10 min, 150 μL of the resultant supernatant was obtained and the concentration of RPG in the sample was determined using the HPLC method.

### 2.4. In Vitro Metabolic Inhibition Study

To construct dose–response curves to determine the inhibitory effect of QCT on the hepatic metabolism of RPG, a microsomal incubation mixture consisting of RLM or HLM (0.5 mg/mL microsomal protein), 1 mM NADPH, 50 mM potassium phosphate buffer, 3 μM RPG, and nine different concentrations of QCT (0, 0.1, 0.5, 1, 5, 10, 50, 100, and 200 μM) were prepared, at a total volume of 200 μL. To construct Dixon plots for the inhibitory effects of QCT on the hepatic metabolism of RPG, five different concentrations of RPG (1, 3, 10, 30, and 100 μM), and five different concentrations of QCT (0, 1, 30, 100, and 200 μM) were used. Microsomal incubation and sample preparation were performed as described in the Section 2.3.

### 2.5. Animals

Sprague-Dawley rats (approximately 250 g) were purchased from Samtako Bio Korea Co. (Gyeonggi-do, South Korea) Rats were housed in ventilated rat cages (Tecniplast USA, West Chester, PA, USA) with access to standard rat chow (Agribrands Purina Canada Inc., Levis, Canada) and water ad libitum, and were allowed to acclimatize for one week prior to the experiments. Protocols for the animal studies were reviewed and approved in accordance with the guidelines of the Institutional Animal Care and Use Committee of Pusan National University (Busan, South Korea; date of approval: 4 May 2020; approval number: PNU-2020-2602).

### 2.6. In Vivo Pharmacokinetic Study in Rats

Rats were anesthetized via intramuscular injection of 10 mg/kg zoletil [35]. The femoral vein and artery of the rats were cannulated using a polyethylene tube (BD Medical, Franklin Lakes, NJ, USA). After recovery from anesthesia, a single intravenous or oral dose (0.4 mg/kg) of RPG with or without a single simultaneous intravenous dose (25 mg/kg) or oral dose (100 mg/kg) of QCT was administered to the rats. The vehicle solutions for the intravenous and oral doses were composed of PEG 400 and aqueous 0.3% CMC solution (50:50 *v*/*v*). Approximately 300 μL of blood was collected via the femoral artery at 0, 1, 5, 15, 30, 60, 90, 120, 180, and 240 min after intravenous injection and at 10, 20, 30, 45, 60, 90, 120, 180, and 240 min after oral administration. After centrifugation of the blood sample at 2000× *g* at 4 °C for 5 min, 120 μL of the resulting supernatant (plasma) was obtained and the concentration of RPG in the sample was determined using the HPLC method.

### 2.7. HPLC Analysis

The concentrations of RPG and QCT in the buffer, microsomes, and (diluted) plasma samples were determined as reported previously [36,37], with slight modifications. For RPG, 50 μL of the sample (or 120 μL of the plasma sample obtained from the in vivo pharmacokinetic study) was deproteinized with 300 μL of acetonitrile that contain ketoconazole (internal standard; 100 ng/mL). For QCT, 200 μL of the microsome sample or diluted plasma was deproteinized with 400 μL of acetonitrile that contained lapatinib (internal standard; 2000 ng/mL). After vortex mixing and centrifugation at 15,000× *g* for 10 min, the resulting supernatant was transferred to a clean 1.5-mL microcentrifuge tube and dried under nitrogen gas at room temperature. The residue was reconstituted with 50 μL of a mobile phase. After vortex mixing and centrifugation at 15,000× *g* for 10 min, 40 μL of the resulting supernatant was injected into the HPLC column (length 250 mm, inner diameter 4.6 mm, particle size 5 μm, pore size 100 Å; Phenomenex, Torrance, CA, USA). For RPG, isocratic elution of a mobile phase consisting of 10 mM phosphate buffer (pH 6.0) and acetonitrile (46.4:53.6, *v*/*v*) was performed at a flow rate of 1 mL/min; the column effluent was monitored by a fluorescence detector (RF-20A; Shimadzu Co., Kyoto, Japan) at 240 nm (λ_ex_)/380 nm (λ_em_) at room temperature. For QCT, gradient elution of a mobile phase consisting of 0.1% TFA in water (solvent A) and acetonitrile (solvent B) was performed at a flow rate of 1 mL/min, and the procedure was as follows (solvent A: solvent B, *v*/*v*): started at 75:25 at 0 min, ramped from 75:25 to 60:40 for 13 min, back to 75:25 for 0.1 min, and maintained for 6.9 min (total run time: 20 min); the column effluent was monitored by an ultraviolet detector (SPD-20A; Shimadzu Co., Kyoto, Japan) at 254 nm at 40 °C. The lower limit of quantitation limit (LLOQ) of the HPLC methods were 10 ng/mL (buffer and plasma samples) and 50 ng/mL (microsome samples) for RPG and 100 ng/mL for QCT. The validation parameters for the HPLC methods were listed in Tables S1 and S2.

### 2.8. Data Analysis

A single-site Michaelis–Menten Equation was simultaneously fitted to the substrate (RPG) concentration ([S]; μM) as follows versus initial metabolic rate (V; pmol/min/mg protein):

(2) $$V=\;\frac{V_{\max}\;\times \;[S]}{K_{m}+[S]}$$

where V_max_ and K_m_ are the maximal metabolic rate and Michaelis–Menten constant, respectively. The intrinsic metabolic clearance (CL_int_) was calculated as V_max_/K_m_. The half maximal inhibitory concentration (IC_50_) of QCT for the hepatic metabolism of RPG was determined via nonlinear regression using GraphPad Prism software (version 5.01; GraphPad Software, San Diego, CA, USA) according to the four-parameter logistic equation:

(3) $$Y=\mathrm{Min}+\frac{\mathrm{Max}\;-\;\mathrm{Min}}{1+(\frac{X}{{\mathrm{IC}}_{50}}{)}^{-P}}$$

where X and Y are the inhibitor concentrations and response, respectively. Max and Min are the initial and final Y values, respectively, and the power P represents the Hill coefficient. The type of inhibition of QCT on the hepatic metabolism of RPG was determined graphically using a Dixon plot. The inhibition constant K_i_ of QCT on the hepatic metabolism of RPG was determined via nonlinear regression using a GraphPad Prism, according to the mixed-model enzyme inhibition equation:

(4) $$\;Y=\;\frac{V_{\max}\;\times \;X}{K_{m}\;\times \;(1+\frac{I}{K_{i}})+X\;\times \;(1+\frac{I}{\alpha \;\times \;K_{i}})}$$

where X, Y, and I are the substrate concentration, enzyme activity, and inhibitor concentration, respectively. V_max_ and K_m_ are the same as those defined in Equation (2). The parameter α is indicative of the inhibition type. The mixed model is a general equation that includes competitive, uncompetitive, and noncompetitive inhibition as special cases. When α = 1, the mixed model is identical to a noncompetitive inhibition. When α is very large (α→∞) or very small (α→0), the mixed model becomes identical to competitive inhibition or uncompetitive inhibition, respectively. In the other cases (α ≠ 1), the mixed model describes mixed inhibition.

Non-compartmental analysis (WinNonlin, version 3.1, NCA200 and 201; Certara, Inc., Princeton, NJ, USA) was conducted to estimate the following pharmacokinetic parameters: total area under the plasma concentration–time curve from time zero to time infinity (AUC); total body clearance (CL, calculated as dose/AUC); terminal half-life (t_1/2_); and apparent volume of distribution at steady state (V_ss_). For comparison, the extent of absolute oral bioavailability (F; expressed as percent of dose administered) was calculated by dividing the dose-normalized AUC after oral administration by the dose-normalized AUC after intravenous injection. The peak plasma concentration (C_max_) and time to reach C_max_ (T_max_) were obtained directly from the measured experimental data.

### 2.9. Statistical Analysis

*p*-values < 0.05 were considered statistically significant. They were calculated using the unpaired *t*-test for comparison between two means or one-way analysis of variance (ANOVA) with post-hoc Tukey’s honestly significant difference test for comparison among three means. Unless indicated otherwise, all data are expressed as the mean ± standard deviation, except for T_max_, which is expressed as median (range), rounded to three significant figures.

## 3. Results

### 3.1. Effects of QCT on the Protein Binding and Blood Distribution of RPG

**Figure 2:**
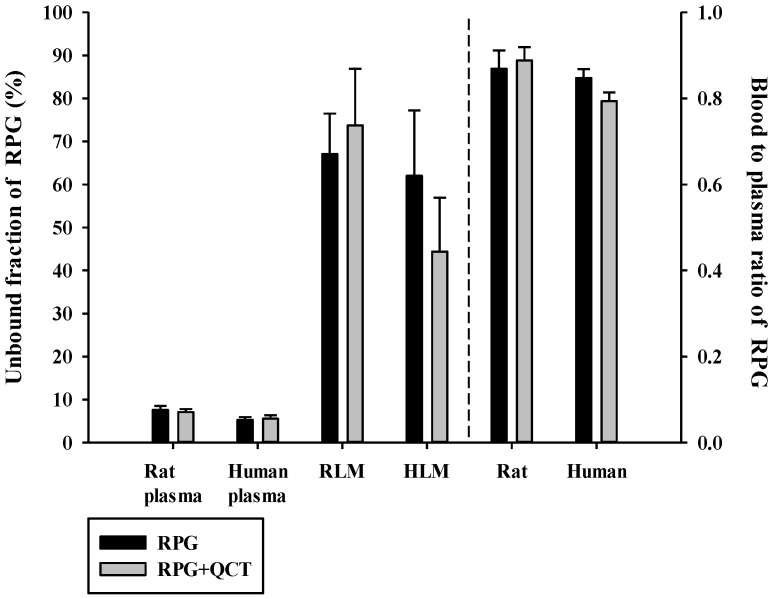
Unbound fractions of RPG in plasma (f_uP_) and liver microsomes (f_uMIC_) in the absence or presence of QCT, and the blood-to-plasma concentration ratios (R_B_) of RPG in rat and human whole blood in the absence or presence of QCT. The rectangular bars and their error bars represent the means and standard deviations, respectively (*n* = 5).

The f_uP_, f_uMIC_, and R_B_ of RPG in the absence and presence of QCT are shown in Figure 2. The f_uP_ of RPG was 0.0533 ± 0.0060 and 0.0761 ± 0.0094 in human and rat plasma, respectively, indicating extensive plasma protein binding. The f_uMIC_ of RPG was 0.620 ± 0.152 and 0.671 ± 0.009 in HLM and RLM, respectively, indicating low to moderate microsomal protein binding. The f_uP_ and f_uMIC_ of RPG were not significantly altered by the presence of QCT (*p* ≥ 0.0609). The rat and human R_B_ of RPG observed in the present study were 0.869 ± 0.043 and 0.847 ± 0.021, respectively, and they were not significantly altered by the presence of QCT (*p* ≥ 0.054). These results indicated minimal effects of QCT on the protein binding and blood distribution of RPG.

### 3.2. Hepatic Microsomal Metabolism of RPG

**Figure 3:**
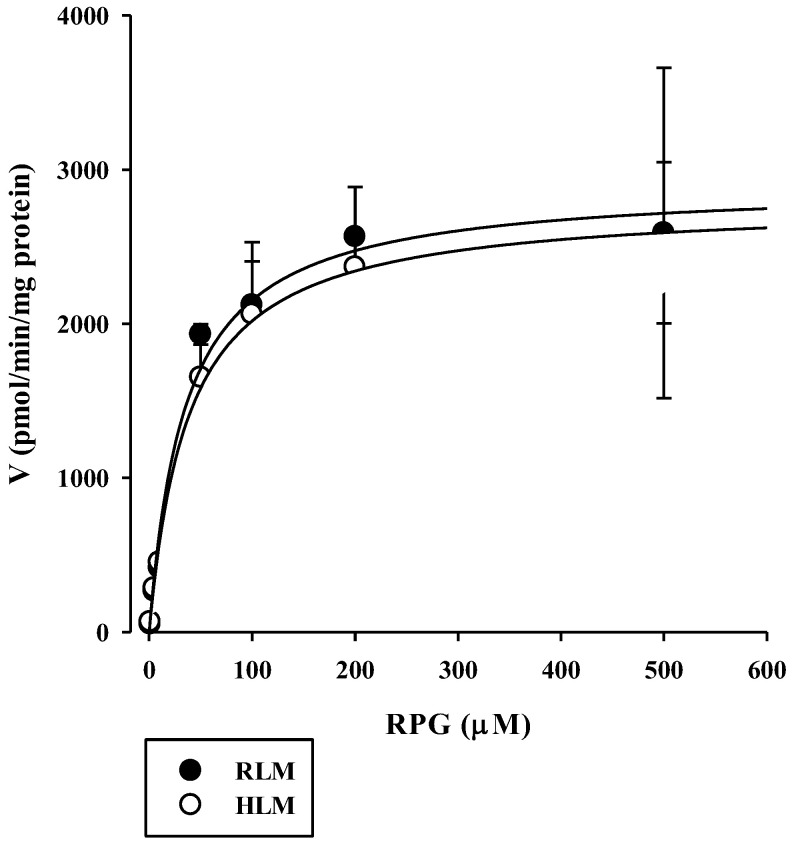
Concentration dependence of the disappearance of RPG in RLM and HLM. The closed circles and their error bars represent the means and standard deviations, respectively (*n* = 4). The solid lines represent the fitted nonlinear regression curves.

The concentration dependence of RPG metabolism in the RLM and HLM was also investigated. As shown in Figure 3, saturable and concentration-dependent metabolic profiles were observed and well-described via Michaelis–Menten kinetics in both RLM and HLM, assuming the presence of one saturable component (*r*^2^ = 0.982–0.996). The V_max_, K_m_, and CL_int_ of RPG in the RLM were estimated to be 1990–4560 pmol/min/mg protein, 16.0–92.2 μM, and 49.5–124 μL/min/mg protein, respectively. The V_max_, K_m_, and CL_int_ of RPG in the HLM were estimated to be 2380–3240 pmol/min/mg protein, 27.0–69.4 μM, and 46.6–89.2 μL/min/mg protein, respectively. There were no significant differences in the metabolic parameters of RPG between RLM and HLM (*p* = 0.656 for V_max_, 0.972 for K_m_, and 0.394 for CL_int_), indicating negligible species differences.

### 3.3. Effects of QCT on the Hepatic Microsomal Metabolism of RPG

**Figure 4:**
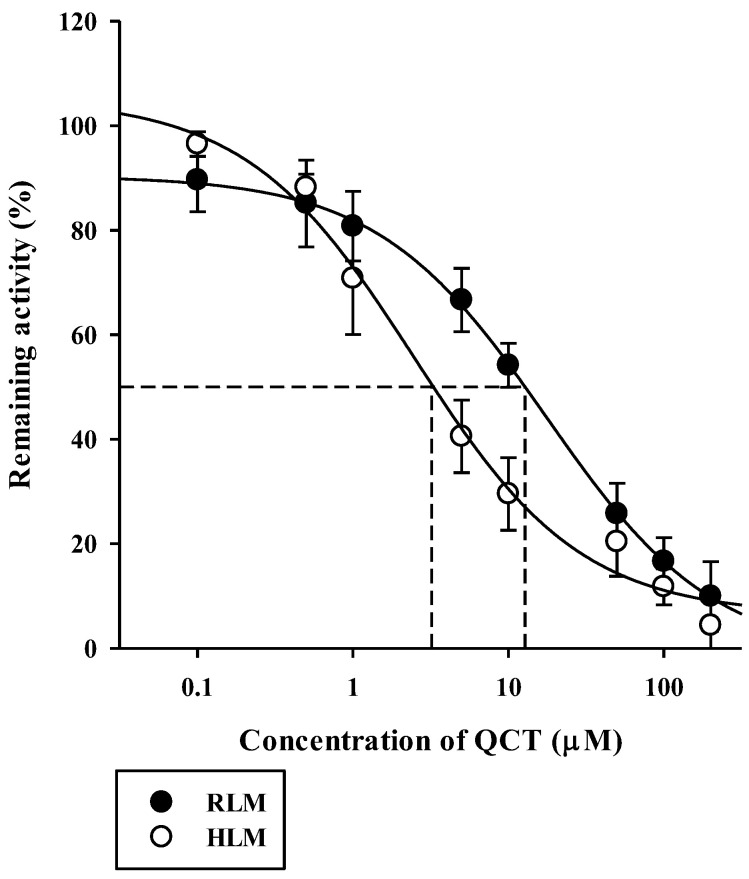
Dose versus response curves for the inhibitory effect of QCT on the disappearance of RPG of 3 μM (well below its K_m_ of 43 μM) in RLM and HLM. The closed circles and their error bars represent the means and standard deviations, respectively (*n* = 4). The solid lines represent the fitted nonlinear regression curves.

**Figure 5:**
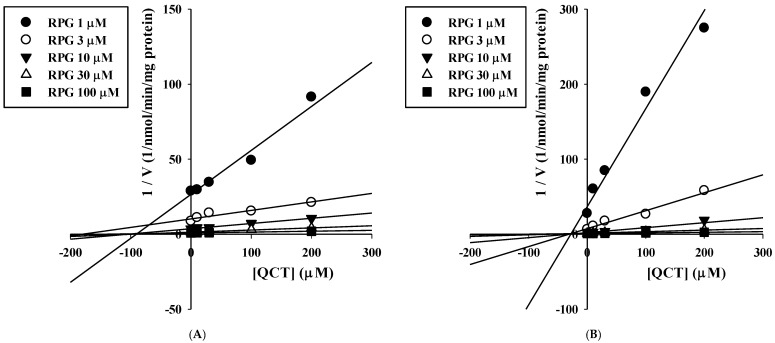
Dixon plots for the inhibitory effects of QCT on the metabolism of RPG in RLM (**A**) and HLM (**B**).

The inhibitory effects of QCT at various concentrations up to 200 μM on the metabolism of QCT in RLM and HLM were assessed by constructing dose–response curves (Figure 4). They were readily described using the sigmoidal logistic equation (Equation (3); *r*^2^ = 0.990–0.999). The mean (range) of the IC_50_ of QCT on the microsomal metabolism of RPG was estimated to be 16.7 (13.0–18.6) μM in RLM and 3.03 (1.53–5.44) μM in HLM (Table 1). The IC_50_ values were significantly lower in the HLM than in the RLM (*p* = 0.000133). The inhibition mechanism of QCT on RPG metabolism was assessed through the construction of Dixon plots. In both RLM (Figure 5A) and HLM (Figure 5B), the plot lines intersected at a point near the *x*-axis in the upper-left quadrant of the plot, indicating mixed inhibition [38]. The K_i_ of QCT on the microsomal metabolism of RPG was estimated by fitting the data to the mixed inhibition model equation (Equation (4)), as listed in Table 1.

### 3.4. In Vivo Intravenous and Oral Pharmacokinetic Studies in Rats

**Figure 6:**
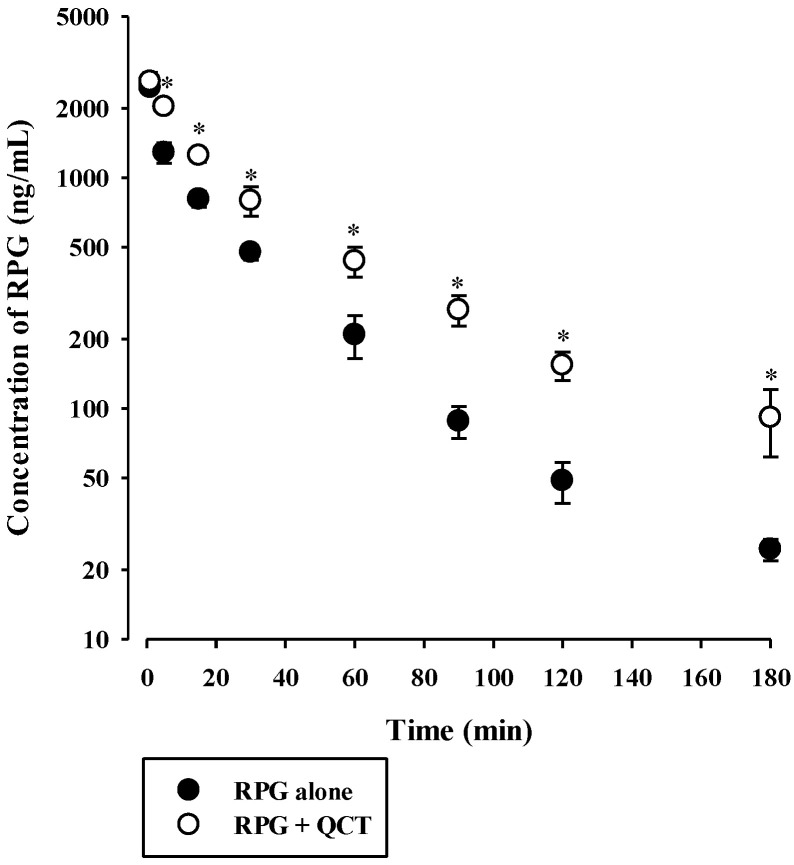
Plasma concentration versus time profiles of RPG following its intravenous administration at 0.4 mg/kg without or with intravenous QCT at 25 mg/kg in rats. The circles and their error bars represent the means and standard deviations, respectively (*n* = 7). The asterisks indicate statistical significance when compared to the control (RPG alone) group (*p* < 0.05).

**Figure 7:**
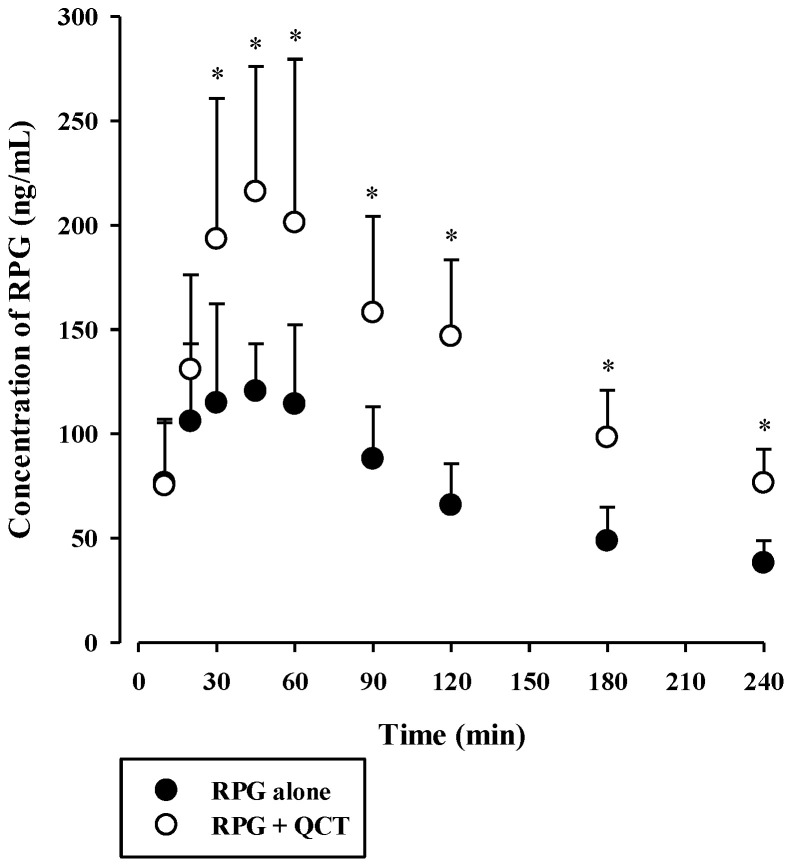
Plasma concentration versus time profiles of RPG following its oral administration at 0.4 mg/kg without or with oral QCT at 100 mg/kg in rats. The circles and their error bars represent the means and standard deviations, respectively (*n* = 7). The asterisks indicate statistical significance when compared to the control (RPG alone) group (*p* < 0.05).

The plasma concentration versus time profiles of intravenous RPG with or without the concurrent administration of intravenous QCT in rats are shown in Figure 6, and the relevant pharmacokinetic parameters are listed in Table 2. The AUC and t_1/2_ of RPG were significantly higher by 83.4% (*p* < 0.0001) and 40.8% (*p* = 0.0022), respectively, whereas its CL was significantly lower by 44.9% (*p* < 0.0001) in rats with concurrent administration of QCT than in control rats. There were no significant differences in the V_ss_ of RPG between the two groups (*p* = 0.061). The plasma concentration versus time profiles of oral RPG with or without the concurrent administration of oral QCT in rats are shown in Figure 7, and the relevant pharmacokinetic parameters are listed in Table 3. The AUC and C_max_ of RPG were significantly higher by 87.6% and 84.2%, respectively, in rats that received a concurrent administration of QCT than in control rats (*p* = 0.000263 and 0.00479, respectively).

## 4. Discussion

This study aimed to systematically investigate the potential metabolic drug–phytochemical interactions between RPG and QCT. A previous study reported that the metabolism of RPG (at 0.2 and 2 μM) in HLM was significantly inhibited by the presence of 25 μM QCT [8]. To our knowledge, however, the present study is the first systematic investigation of the kinetic mechanisms of the inhibitory actions of QCT on the metabolism of RPG and their possible species differences. Since CYP2C8- and CYP3A4-mediated metabolism are principally responsible for the elimination of RPG in humans [7,8], the results of the present in vitro microsomal metabolic interaction studies can be interpreted and discussed based on the principles and guidelines of CYP-mediated drug–drug interactions. The apparent K_i_ values represent the dissociation constant for the interaction between the inhibitor and the enzyme [39]. As the concentration of the inhibitor (QCT) increased from 0 μM to 200 μM in the present K_i_ estimation study, the V_max_ of RPG metabolism tended to decrease (RLM: 2016 to 928 pmol/min/mg protein; HLM: 1507 to 818 pmol/min/mg protein) and the K_m_ of RPG metabolism tended to increase (RLM: 53 to 81 μM; HLM: 23 to 111 μM). These results are typical diagnostic signatures of mixed inhibition [40], which are consistent with the graphical analysis of the constructed Dixon plots (Figure 5). A mixed inhibitor can bind to the enzyme–substrate complex as well as free enzymes, but it has a higher affinity for one state than the other [41]. In our present results, the α value (representing the extent to which the binding affinity between enzyme and substrate is changed by the inhibitor) was much greater than 1, implying that the inhibitor (QCT) may bind with a higher affinity to the free enzyme (CYP2C8/3A4) than the enzyme-substrate (RPG) complex [42]. Collectively, it is plausible that QCT can inhibit the hepatic metabolism of RPG in vitro via a mixed mechanism and the inhibition potency is weak in rats and moderate in humans.

To investigate the in vivo consequences of the aforementioned in vitro metabolic inhibition data, the pharmacokinetics of RPG with or without concurrent administration of QCT was assessed in rats. The in vivo intravenous and oral doses of RPG and QCT were selected based on previous rat pharmacokinetic studies [43,44,45,46]. In our present rat study, the urinary and fecal excretion of RPG administered intravenously was negligible (≤1.60% of the dose) [36], suggesting that RPG is primarily eliminated via metabolic routes. Assuming that RPG is metabolized exclusively by the liver, the hepatic clearance (CL_H_) of RPG becomes equivalent to its blood clearance (calculated as CL/R_B_ = 9.11 mL/min/kg). Thus, the hepatic extraction ratio (E_H_) of RPG can be estimated to be 0.114–0.182, by dividing the CL_H_ by the rat hepatic blood flow (Q_H_, 50–80 mL/min/kg) [47]. The CL_H_ of a drug with a low E_H_ primarily depends on its unbound fraction in the blood (f_B_) and CL_int_, based on the well-stirred hepatic clearance model. Because the f_B_ (=f_P_/R_B_) of RPG was not significantly altered by the presence of QCT, the reduced CL of intravenous RPG by the co-administration of QCT (Table 1) could be attributed to a decrease in the CL_int_ of RPG, resulting from the observed inhibitory effects of QCT on RPG metabolism.

The F of RPG was known to be approximately 62.5% in humans [6], which coincides closely with that observed in the present rat study (Table 3). F can be determined by the fraction absorbed (F_abs_), intestinal availability (F_G_), and hepatic availability (F_H_) as follows: F = F_abs_ × F_G_ × F_H_ = F_abs_ × (1 − E_G_) × (1 − E_H_), where E_G_ is the GI extraction ratio. Consistent with intravenous RPG, the fecal excretion of oral RPG was observed to be minimal (2.21–5.78% of the dose administered). Thus, F_abs_ can be assumed to be 1, and the F_H_ of RPG in rats can be estimated to be 0.818–0.886 from our present rat data. The F_G_ of RPG can be calculated to be 0.572–0.620 in rats using the equation mentioned above, suggesting that considerable gut first-pass effects of RPG may occur, which warrants further investigation. Thus, in our present oral study using a combination of RPG and QCT, it is plausible that the gut and hepatic first-pass and hepatic systemic metabolism of RPG could have been inhibited by QCT, resulting in the increased systemic exposure (AUC and C_max_) of RPG.

Notably, oral administration of QCT increased the AUC of orally administered RPG by 1.88-fold, indicating that QCT can be classified as an weak inhibitor of in vivo metabolism and systemic exposure of RPG in rats [48]. This suggests that a single oral QCT dose of 100 mg/kg achieved GI and hepatic QCT levels sufficient to significantly inhibit the first-pass and systemic metabolism of RPG in vivo. Previous clinical studies have reported controversial results regarding the pharmacokinetic interactions between QCT and therapeutic drugs. In healthy subjects, oral treatment with QCT at 500 mg/day for 21 days did not significantly change the pharmacokinetics of rosiglitazone [49], whereas the systemic exposure (AUC and/or C_max_) of chlorzoxazone and caffeine were significantly increased by oral treatment with QCT at 1000 mg/day for 10 days and 500 mg/day for 14 days, respectively [14,50]. Based on the FDA guidelines [51], the magnitude of in vivo clinical herb–drug interactions between RPG and QCT was predicted from the in vitro protein binding and metabolism data. The ratio of the AUC of RPG in the presence and absence of QCT was predicted to be 1.03–1.54 by the basic (simple static) model (for a detailed calculation process, see Table S3 in the Supplementary Information). This suggests that QCT could act as a significant inhibitor for RPG metabolism in clinical settings, and there is a possibility that clinically significant pharmacokinetic interactions between QCT and RPG could occur, depending on the extent and duration of QCT intake from food products and dietary supplements.

## 5. Conclusions

This study clearly indicates that QCT can inhibit the hepatic metabolism of RPG in vitro via a mixed mechanism. Furthermore, the in vivo systemic exposure of RPG following intravenous and oral administration in rats was significantly increased by the concurrent administration of QCT. Based on the FDA guidelines, the magnitude of in vivo clinical herb–drug interactions between RPG and QCT was predicted to be a 1.03–1.54-fold increase in the AUC of RPG. These results suggest that clinically significant pharmacokinetic interactions between QCT and RPG could occur, which warrant further systematic clinical investigation.

## Figures and Tables

**Table 1:** Enzyme kinetic parameters for the metabolism of RPG and its inhibition by QCT in RLM and HLM.

Parameter	RLM	HLM
Metabolism of RPG		
V_max_ (pmol/min/mg protein)	3070 ± 960	2850 ± 417
K_m_ (μM)	43.3 ± 29.7	42.8 ± 16.7
CL_int_ (μL/min/mg protein)	85.5 ± 29.2	71.8 ± 17.7
Inhibition of RPG metabolism by QCT		
IC_50_ (μM)	16.7 ± 2.6	3.03 ± 1.84 *
K_i_ (μM)	72.0	24.2
α	2.88	14.4
Type	Mixed	Mixed

* Significantly different from the ‘RLM’ group (*p* < 0.05).

**Table 2:** Pharmacokinetic parameters of RPG following its intravenous administration at 0.4 mg/kg without or with simultaneous intravenous administration of QCT at 25 mg/kg in rats (*n* = 7).

Parameter	RPG alone	RPG with QCT
AUC (μg·min/mL)	50.7 ± 3.0	92.9 ± 11.8 *
t_1/2_ (min)	40.8 ± 4.2	57.4 ± 10.6 *
CL (mL/min/kg)	7.92 ± 0.47	4.36 ± 0.54 *
Ae_U_ (% of dose)	1.60 ± 0.77	1.32 ± 0.88
Ae_GI_ (% of dose)	ND	ND
V_ss_ (mL/kg)	293 ± 18	269 ± 25

* Significantly different from the control (RPG alone) group (*p* < 0.05).

**Table 3:** Pharmacokinetic parameters of RPG following its oral administration at 0.4 mg/kg without or with simultaneous oral administration of QCT at 100 mg/kg in rats (*n* = 7).

Parameter	RPG Alone	RPG with QCT
AUC (μg·min/mL)	25.7 ± 6.6	48.2 ± 9.6 *
t_1/2_ (min)	139 ± 66	148 ± 49
C_max_ (ng/mL)	129 ± 33	238 ± 76 *
T_max_ (min)	45 (30–45)	45 (30–60)
Ae_U_ (% of dose)	0.458 ± 0.441	0.371 ± 0.336
Ae_GI_ (% of dose)	3.41 ± 1.65	2.90 ± 2.50
F (%)	50.7	95.1

* Significantly different from the control (RPG alone) group (*p* < 0.05).

## Data Availability

The data presented in this study are available in the paper.

## Supplementary Materials

The following are available online at https://www.mdpi.com/article/10.3390/pharmaceutics13060782/s1, Figure S1: Cornish-Bowden plots for the inhibitory effects of QCT on the metabolism of RPG in RLM and HLM, Table S1: Within-run and between-run precision and accuracy of the present bioanalytical method for the quantification of RPG in biological matrices, Table S2: Within-run and between-run precision and accuracy of the present bioanalytical method for the quantification of QCT in biological matrices, Table S3: Parameters related to the estimation of R value. References [51,52] are cited in the supplementary materials.
