# Supplementary material for: Assessment of Metabolic Interaction between Repaglinide and Quercetin via Mixed Inhibition in the Liver: In Vitro and In Vivo
Source: Pharmaceutics. 2021 May 23;13(6):782. doi: 10.3390/pharmaceutics13060782 (PMC8224802; doi:10.3390/pharmaceutics13060782)
Supplement: Supplementary file 1 [file pharmaceutics-13-00782-s001.zip › pharmaceutics-1167130-supplementary.pdf]

pharmaceutics

MDPI

Supplementary Materials: Assessment of Metabolic Interaction between Repaglinide and Quercetin via Mixed Inhibition in the Liver: In Vitro and In Vivo

Ji-Min Kim, Seong-Wook Seo, Dong-Gyun Han, Hwayoung Yun and In-Soo Yoon

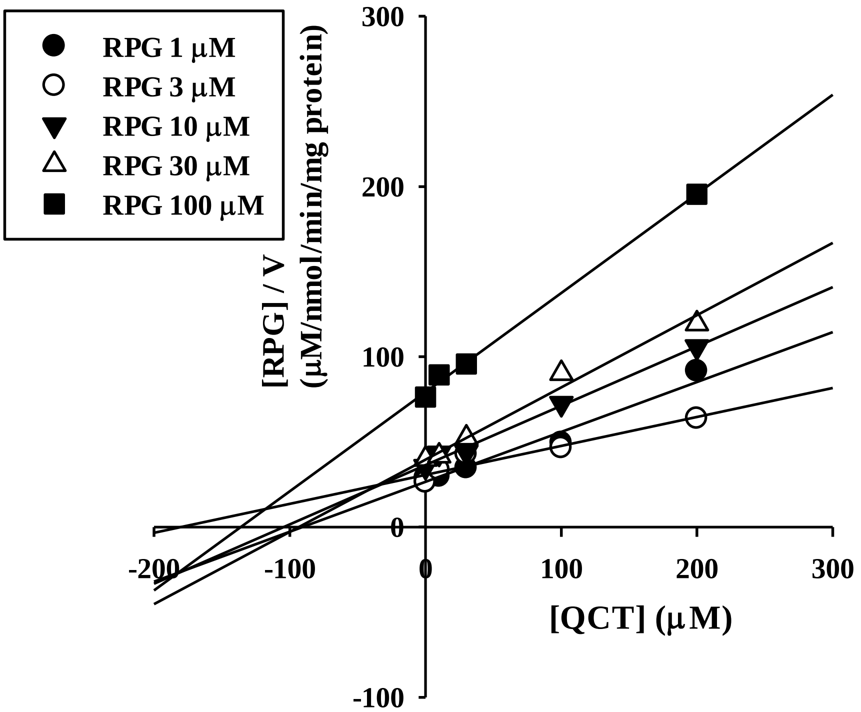

(a)

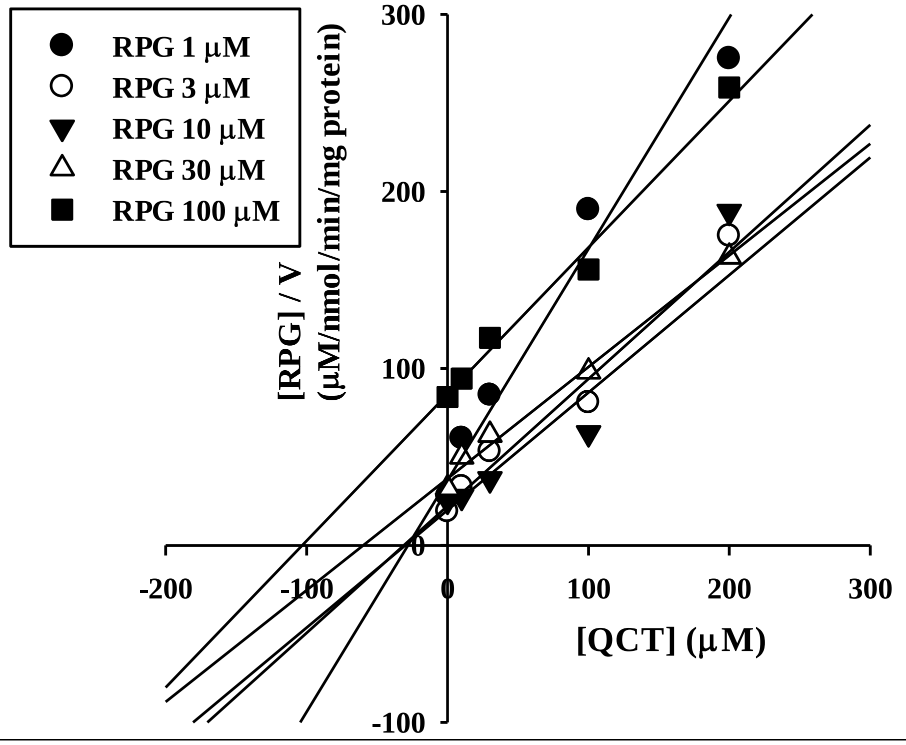

(**b**)

Figure S1. Cornish-Bowden plots for the inhibitory effects of QCT on the metabolism of RPG in RLM (**a**) and HLM (**b**).

Table S1. Within-run and between-run precision and accuracy of the present bioanalytical method for the quantification of RPG in biological matrices (n = 3).

Nominal Concentration (ng/mL)	Precision (%)		Accuracy (%)	
	Within-run	Between-run	Within-run	Between-run
Plasma				
LLOQ (10)	3.41	2.54	97	101
LQC (30)	6.99	6.62	96	105
MQC (120)	8.74	4.90	98	98
HQC (1200)	1.93	5.31	105	99
Urine				
LLOQ (10)	5.74	5.77	99	100
LQC (30)	2.20	4.67	106	106
MQC (120)	2.23	2.82	108	107
HQC (1200)	6.65	6.60	97	99
GI				
LLOQ (10)	8.82	7.08	100	103
LQC (30)	5.01	5.37	103	105
MQC (120)	3.66	2.17	108	109
HQC (1200)	5.03	5.02	97	99
PBS				
LLOQ (10)	6.17	4.51	102	100
LQC (30)	6.70	5.66	102	100
MQC (120)	5.30	2.35	100	101
HQC (1200)	1.36	1.42	101	100
HLM				
LLOQ (50)	4.94	3.96	99	99
LQC (150)	1.84	6.86	106	102
MQC (600)	4.52	3.62	106	100
HQC (1200)	1.16	2.94	105	102

RLM				
LLOQ (50)	4.25	3.56	104	108
LQC (150)	1.98	1.92	102	102
MQC (600)	8.68	1.08	100	101
HQC (1200)	3.48	3.82	104	98

Table S2. Within-run and between-run precision and accuracy of the present bioanalytical method for the quantification of QCT in biological matrices ($n = 3$).

Nominal Concentration (ng/mL)	Precision (%)		Accuracy (%)	
	Within-run	Between-run	Within-run	Between-run
Plasma				
LLOQ (100)	2.97	1.21	106	106
LQC (300)	3.66	5.82	99	99
MQC (1600)	3.65	5.00	107	104
HQC (8000)	0.56	0.37	105	106
PBS				
LLOQ (100)	5.42	4.90	92	92
LQC (300)	2.29	4.69	108	104
MQC (1600)	7.71	6.34	103	105
HQC (8000)	5.38	2.72	108	109

Table S3. Parameters related to the estimation of R value.

Parameter	Value	Description & Source
$f_{u,QCT}$	0.0462	In-house
$f_{u,mic,QCT}$	0.381	In-house
IC_{50} (μM)	1.66	Figure 4
S (μM)	5	Figure 3
K_m (μM)	27.02	Figure 3
Observed K_i (μM)	8.712	Figure 5
Calculated K_i (μM)	0.534	$(f_{u,mic,QCT} \times IC_{50}) / (S / K_m + 1)$
$I_{max,u}$ (μM)	0.290	[1]
R	1.03–1.54	$1 + I_{max,u} / K_i$

Based on the FDA guideline [2], the magnitude of in vivo clinical herb-drug interaction (HDI) between RPG and QCT was predicted using the basic (simple static) model (Equation (S1)), assuming that RPG is eliminated exclusively by hepatic CYP-mediated metabolism and QCT acts as a reversible inhibitor for RPG metabolism.

$$R = 1 + I_{max,u} / K_i \quad \text{(S1)}$$

where R is the predicted ratio of the AUC of RPG in the presence and absence of QCT, $I_{max,u}$ is the maximum unbound plasma concentration of QCT, and K_i is the unbound inhibition constant determined in vitro or estimated by the following equation (Equation (S2)). The FDA guideline indicates that, if $R \geq 1.02$, the sponsor should further investigate the DDI potential by either using mechanistic models or conducting a clinical DDI study with a sensitive index substrate.

$$K_i = IC_{50} / (S / K_m + 1) \quad \text{(S2)}$$

where S is the fixed concentration of RPG, and K_m is the Michaelis-Menten constant. The parameters relevant to the estimation of R value are listed below. Based on the estimated R value, it is plausible that QCT could acts as a clinically significant inhibitor for RPG metabolism, suggesting the HDI potential between RPG and QCT in clinical setting, which warrants further in vivo clinical investigation.

References

1. Ganio, M. S.; Armstrong, L. E.; Johnson, E. C.; Klau, J. F.; Ballard, K. D.; Michniak-Kohn, B.; Kaushik, D.; Maresh, C. M. Effect of quercetin supplementation on maximal oxygen uptake in men and women. *J. Sports Sci.* **2010**, *28*, 201–208.

2. Center for Drug Evaluation and Research. In Vitro Drug Interaction Studies: Cytochrome P450 Enzyme- and Transporter-Mediated Drug Interactions. *USA Food and Drug Administration Guidance for Industry.* **2020**, Available online: https://www.fda.gov/media/134582/download. (Accesed date: 1/5/2021)
